# The role of women in promoting voluntary medical male circumcision uptake: Literature review

**DOI:** 10.4102/hsag.v27i0.1794

**Published:** 2022-07-25

**Authors:** Grace Danda, Thandisizwe Mavundla, Christina Mudokwenyu-Rawdon

**Affiliations:** 1 Department of Nursing and Midwifery Sciences, National University of Science and Technology, Bulawayo, Zimbabwe; 2 Department of Nursing Education, University of the Witwatersrand, Johannesburg, South Africa; 3 Freelance Midwifery Consultant, Harare, Zimbabwe

**Keywords:** voluntary medical male circumcision (VMMC), social support systems, VMMC uptake, role of women, HIV prevention

## Abstract

Voluntary medical male circumcision (VMMC) is a global strategy for reducing female-to-male sexual transmission of HIV. Women whose partners are circumcised benefit from a reduced risk of contracting other sexually transmitted diseases; making their role in VMMC critical. The objective of our study was to identify and synthesize existing evidence related to women’s role in promoting VMMC from a regional perspective. The review and selection process were guided by the Problem; Intervention; Comparison and Outcome (PICO) model, which facilitated the exclusion of irrelevant studies. The search strategy search terms for the PICO components with synonyms, related terms and specialist terms were harvested from the Medical Subject Headings (MeSH)© and Embase©. The inclusion criteria were published studies in English and relevant to women’s role in VMMC for the prevention of HIV between 2007 and 2020. Four key categories emerged from the literature as follows: role of women, VMMC uptake, barriers and facilitators of VMMC. The majority of the studies concur on the importance of involving women in VMMC uptake as they have the power to negotiate with their male partners through communication and can persuade men to be circumcised, making it a joint decision. The benefits of VMMC in improving sexual pleasure and attractiveness of the penis seemed to positively convince women to influence and educate men to improve the uptake of VMMC. Women are motivated to convince men to undergo male circumcision (MC) because of the benefits associated with them such as reduction of HIV transmission and cervical cancer. There are, however, limited studies focusing on women’s involvement in VMMC; hence, more research to explore this area is recommended.

**Contribution:**

This review revealed the important role played by women in influencing men to undergo MC but highlight the need for more studies on women’s involvement in VMMC.

## Introduction

The HIV preventive strategy of male circumcision (MC) was launched in 2009 with an expected coverage of 80% by 2016, meaning 20.8 million circumcisions in the 14 priority countries (Uganda, South Africa, Kenya, Lesotho, Zimbabwe, Malawi, Mozambique, Tanzania, Namibia, Zambia, Rwanda, Ethiopia, Swaziland and Botswana) (WHO 2012). These 14 countries were prioritised by WHO because of their high HIV prevalence and low VMMC uptake. According to the UN statistics, the prevalence of HIV gradually declined globally from 2.1 million in 2010 to 1.7 million in 2018. This left the world far below the expected to less than 500 000 new infections by 2020. Sub-Saharan Africa has been seen to be the most affected in the world with an HIV prevalence rate of 69% and an MC prevalence rate of 30%. Zimbabwe, being among the 14 priority countries, has seen a slight decline in HIV prevalence from 13.8 in 2015 to 12.7 in 2018; with an MC prevalence rate of 10%. Zimbabwe has had challenges in achieving the expected target since 2009 when MC was introduced in the country despite various approaches used to facilitate MC uptake, which include posting adverts in various platforms online or going to schools and using different materials to promote the programme (UNAIDS DATA 2019).

According to a study by Macintyre et al. (2014:e83998) on VMMC uptake, the influence by family and community relationships was seen to impact men’s decision to circumcise, hence the need to focus on women’s role in VMMC because the area has not been investigated adequately. Furthermore, a study carried out in three African countries (South Africa, Zimbabwe and Tanzania) realised that young women influenced the decision by their partners to take up MC both directly and indirectly. The indirect influence by most female participants in Tanzania and Zimbabwe was through not initiating relationships or readily discontinuing them if their partners refused VMMC. A few female participants, however, mentioned directly using the threat of infections, HIV and cervical or penile cancer as a means of persuasion. Other participants felt that a woman should show her genuine concern by convincing her partner to have MC, regardless of benefits for herself (Kaufman et al. 2018:5186). Role is defined as the function assumed or part played by a person or a thing in a particular situation. Merriam Webster dictionary (2017) further explained the role as ‘a socially expected behaviour pattern usually determined by an individual’s status in a particular society’. The role of women in this study was determined by a broad literature search focusing on empirical studies performed on social support systems in VMMC uptake:

[*W*]omen play an important part in influencing male circumcision uptake. Women have been shown to influence and make decisions about whether their sons are circumcised as well as sway their male sexual partner’s decision to become circumcised. (Rain-Taljaard et al. 2003:316)

A study on adolescent and adult males seeking VMMC in Uganda found that those who were in a relationship or were married had been influenced by their female partner to seek VMMC (Lunsford et al. 2017:40). The men viewed women as holding negotiating power when communicating with their male partners and being likely to persuade men to be circumcised, making it a joint decision (Lanham et al. 2017; Reiss, Achieng & Bailey 2014:e97748):

[*W*]omen can also be a source of information about MC for their male partners, and there is evidence that a woman’s preference for a circumcised partner is influencing male interest in circumcision. (Baeten et al. 2010:1190–1197; Reiss et al. 2014:e97748)

Another study by Chikutsa and Muharaj (2015:603) on social representations of MC observed the importance of women involvement in VMMC. A view came out that women have persuasive power and thus can influence their sons and husbands to get circumcised. Even after the circumcision has been performed, women do have a counselling role during the healing process.

A study by Mazambara, Hlungwani and Nyembezi (2017) in Zimbabwe on perceptions and experiences of female participants on VMMC observed that the main role of women pre-VMMC was to encourage their partners to go for MC and post-VMMC to be that of counselling. Some participants in the same study assumed the importance of awareness on MC in women to encourage men. Layer et al. (2014:259) asserted that women can take advantage of their position in a relationship to negotiate for VMMC to their partners. Women are able to either dissuade or persuade their partners to take up MC, hence the importance of utilising their ability in creating demand for the programme (Glick 2014).

The roles of women are outlined in the forms of support they provide to their partners and sons according to Cutrona and Suhr (1992:154–174) who stated five categories in the definition of social support as either being emotional, tangible, informational, social network or esteem.

The knowledge provided in the form of advice or facts is a form of *informational support*. This comes in handy when women encourage men to undergo circumcision during couple discussion. When women reach out to men, this assists to promote uptake of MC as proven in behaviours for protection from HIV transmission, where women play a key role. As couple communication is already in place, it is beneficial for the VMMC programmes to take its advantage in order to promote MC. In this category, women create awareness to men about VMMC, educate and talk to men about VMMC.

Expressing empathy, concern, sympathy or caring are all forms of *emotional support* and wives, mothers and partners provide hope and a listening ear. Emotional support comes in during pre- and post-MC stage when the female partner counsels her nervous, jittery and emotional partner in anticipation of the pending medical procedure and after the procedure for support on care of the wound and abstinence.

*Companionship support*, also known as esteem support, or regard, reverence, honour, approval, respect; or appraisal defined as assessment, review, evaluation or judgement. To promote (indorse, endorse, encourage, help, sponsor) one’s intrinsic value, abilities and skills are all forms of companionship support. It is also known as friendship (bond, relationship, alliance, attachment, acquaintance), camaraderie (friendship, amity, companionship, solidarity and company) and comradeship (friendship, amity, companionship, solidarity and company). Men are influenced, swayed or persuaded by women to undergo MC.

Some ways of *social network support* include facilitating a sense of belonging to a group with situations or interests similar to theirs. A woman’s commitment to her partner is shown by supporting him through accompanying him to VMMC services and counselling. However, other women feel MC is ‘a man’s thing’, and that it reflects badly if a female partner escorts him for VMMC. The woman can, however, assist the partner to abstain, accompany him for reviews, and ensure he gets a healthy diet and assisting him in wound care to show her post circumcision support.

Another form of support is *tangible support* when women provide services and goods to their partners. Tangible support (touchable, palpable, perceptible, concrete and physical) is also called instrumental support (contributory, active, involved, helpful and influential). This form of social support encompasses the concrete, direct ways where female partners assist their male partners, which can be in the form of insisting that men take up VMMC by explaining the benefits and assisting the men to take care of the wound postoperatively.

Surveys carried out on VMMC have proved that awareness about VMMC among men is quite high but women need to be more involved to assist in convincing their husbands on the importance of the intervention. Some reasons given by older men for not circumcising were that their partners refused, this calls for importance of creating MC awareness to women (Kobayashi 2014). A study by Jasi and Mapingure (2014) on predictors for MC showed that significant determinants of VMMC uptake in Zimbabwe included social support, self-efficacy and availability of services.

No studies that were reviewed show evidence of use of a framework to empower women to be able to support men in taking up MC.

## Research methodology

### Search strategy

The review and selection process were guided by PICO (Cooke, Smith & Booth 2012:1435–1443), which facilitated the exclusion of irrelevant studies.

The search strategy search terms for the PICO components with synonyms, related terms and specialist terms were harvested from the Medical Subject Headings (MeSH)© and Embase©. Both primary (grey literature) and secondary sources were included in the search: PubMed, Google Scholar, EBSCO-CINAHL, Dimensions, Web of Science, SCOPUS, the Cochrane Library of Systematic reviews and African Journals Online (AJOL). The researchers identified studies performed in Africa on VMMC through a narrative review from January 2017 to January 2021. The inclusion criteria were published studies in English and relevant to women’s role in VMMC for the prevention of HIV between 2007 and 2020.

A total of 1500 records were identified in the initial database search. An additional 120 records were identified through other means such as the library repositories and hard copy documents. Further refining was performed where duplicates and unrelated articles were removed and 150 records remained. Of the 150, 100 were excluded because of failure to get full manuscripts and some oversights having been overlooked like the specificity to women in VMMC. Of the 50 remaining records, 22 were finally excluded as the researchers identified some which still were general to VMMC and some excluded using the Critical Appraisal Skills Programme (CASP) checklist, an appraisal tool used to assess quality for the studies. Finally, 28 articles were acceptable and met the criteria for evaluation according to the CASP checklist.

The PICO literature review summary is elaborated in the following table (Table 1), to guide the search of literature review and to exclude the irrelevant literature. The acronym PICO stands for P-population, patient or problem (role of women in VMMC); I - Intervention (promote VMMC uptake); C - Comparison or control (barriers to VMMC) and O - Outcome (benefits of VMMC) as linked here with the literature reviewed.

**FIGURE 1:**
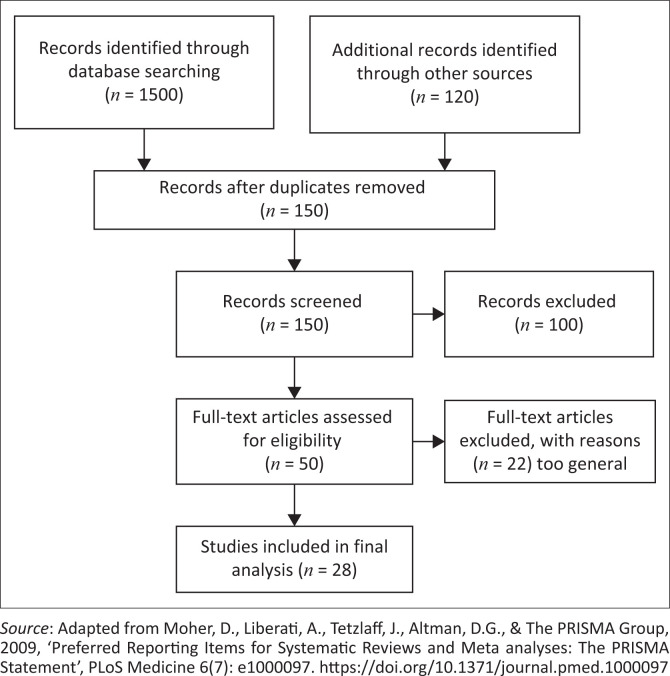
Literature review flow chart guided by the PICO model.

**TABLE 1:** The PICO literature review.

PICO element	PICO concept description	Design	Country
Population, patient or problem Role of women in VMMC uptake	Bangidza (2014): Women must have adequate knowledge on male circumcision if they are to execute their role of encouraging and convincing their partners to take up circumcision services.	Qualitative	Zimbabwe
	Chilungo et al. (2015): Social networks centred on the wives and non-marital partners who encourage men to go for VMMC. Recommended development of messages that specifically address women on the benefits of VMMC.	Mixed	Malawi
	Rupfutse et al. (2014:337): Having a friend who is circumcised and encouragement by a friend or relative or a female partner was a strong facilitator for VMMC.	Quantitative	Zimbabwe
	Layer et al. (2014:258–272); Reiss et al (2014:e97748): Encouragement of men by women was by either making a precondition to start a sexual relationship by refusing to have sexual relations until they undergo male circumcision.	Qualitative Qualitative	Tanzania Kenya
	Kobayashi (2014): Women may influence their male sexual partners’ decision to be circumcised; most people have correct knowledge about VMMC, but there was a gap between knowledge and behaviour. Men motivated by social support from friends were noted to have three times higher chances of circumcision compared to those without the support.	Mixed	Zimbabwe
	Wirth et al. (2016:1007–1013): Influence of female partners played an important positive role in men to become circumcised. The primary reason for women to encourage their partners to undergo VMMC was the protection from cervical cancer when partners are circumcised.	Mixed	Botswana
	Lunsford et al. (2017:39–46): A joint decision was identified when women would negotiate with their partners through communicating and persuading them to be circumcised.	Qualitative	Uganda
	Reiss et al. (2014:e97748): Supported the issue of influence by women for men to undergo VMMC. Talking and insisting to men to be circumcised was a way of influencing men by women.	Qualitative	Kenya
	Chikutsa and Muharaj (2015:603): Importance of women involvement in VMMC, influence of husbands and sons by women is facilitated through their power of persuasion.	Qualitative	Zimbabwe
	World Bank meta-analysis: Gender balance is assisted by educating females to empower them to negotiate, which also improves women’s health status.	Qualitative	Papua New Guinea
	Mazambara et al. (2017): Women are influential in male circumcision decision making by their partners and sons.	Mixed	Zimbabwe
Intervention Promote VMMC uptake	Westercamp et al. (2009): Women advocate for their partners to be circumcised, prefer circumcised men and were forthcoming to have their sons circumcised.	Mixed	Kenya
	Reiss et al. (2014:e97748): Since women have been noted to prefer circumcised partners, this works as a form of influence to men when women give information to their partners about male circumcision.	Qualitative	Kenya
	Jones et al. (2014:278–284); Cook et al (2016): When a female discusses with her partner about male circumcision, this assists to determine the readiness of men for VMMC.	Mixed	Zambia
	Cook et al. (2016:2503–2513): It is recommended that female partners be included in male circumcision promotion since the acceptance of the programme by women has been seen to positively impact men’s decision for male circumcision.	Quantitative	Zambia
	Maguwu (2010): Recommended involvement of women as partners of men who need circumcision to ensure success of the public health promotion. Most men indicated that they would accept to undergo VMMC if approached by their sexual partner rather than if they were approached by a service provider.	Quantitative	Zimbabwe
	Humphries et al. (2014:920–931): Issues of sexual appeal seem to have an impact on deciding to undergo male circumcision. Most men felt that women play an indirect role in influencing men to decide to undergo male circumcision.	Qualitative	South Africa
	Kaufman et al. (2018:5183–5188): For men who refuse VMMC, women felt that they would not start a relationship with them or would quickly end such a relationship because they would put them at high risk of infections like HIV or cervical cancer.	Qualitative	Tanzania and Zimbabwe
	Nxumalo and Mchunu (2019:9–17): If women are knowledgeable, they readily accept male circumcision and can motivate men to undergo the procedure.	Systematic review	Sub-Saharan Africa
	Jasi and Mapingure (2014): Availability, social support and self-efficacy.	Mixed	Zimbabwe
Comparison or context Reduced VMMC uptake Barriers of VMMC	Plotkin et al. (2013:108–116): Culturally, it is believed that men should be circumcised before adolescence and becomes a shame for one to be circumcised when older and having challenges associated with abstaining.	Mixed	Tanzania
	Taruvinga (2014): Barriers of undergoing circumcision include myths and misconceptions about the procedure, partners refusing to have their men circumcised, some are afraid of pain or HIV testing.	Quantitative	Zimbabwe
	Rupfutse et al. (2014:337): Some men were deterred from circumcising because they were afraid of pain and being unsure about how the wound will heal.	Quantitative	Zimbabwe
	Chikutsa and Muharaj (2015:603): Promiscuity suspicions defeat the whole purpose of VMMC to promote HIV prevention. Kobayashi (2014): Barriers were partner refusal because of lack of trust, misconceptions and being afraid of pain.	Qualitative	Zimbabwe
Outcome Benefits of VMMC Facilitators of VMMC	Cook et al. (2016:2503–2513): Reduces chances of sexually transmitted infections and conditions such as phimosis, paraphimosis and posthitis.	Quantitative	Zambia
	Plotkin et al. (2013:108–116): Factors influencing uptake of VMMC included protection from HIV and other STIs.	Mixed	Tanzania
	Reiss et al (2014:e97748): Lower risk of contracting HIV and STIs, women preferred circumcised men because of the hygiene associated with VMMC, taking longer to ejaculate and providing protection from STI/HIV.	Qualitative	Kenya
	Humphries et al. (2015:920–931): There is the belief that circumcision increases sexual performance, partner satisfaction, enlargement of the penis and being able to have several partners.	Qualitative	South Africa
	Wirth et al. (2016:1007–1013): Improved hygiene and cleanliness; increased sexual pleasure, attractive penis and assumption that women love men who are circumcised. Other benefits include cervical cancer protection to women and reduction of HIV transmission, which all contribute to the decision to undergo male circumcision.	Mixed	Botswana
	Chikutsa and Muharaj (2015:603): Satisfy a woman, stronger erection	Qualitative	Zimbabwe
	Kaufman et al. (2018): Protection reduces chances of acquiring HIV and it is hygienic and makes the penis attractive; sex is more pleasurable with a circumcision that can prolong sex.	Qualitative	Tanzania and Zimbabwe
	Mazambara et al. (2017): Benefits of VMMC were hygiene, protection from HIV/STIs, better sexual performance and improved relationships. There were some misconceptions such as no need to use condoms after circumcision.	Mixed	Zimbabwe
	Chilungo et al. (2015): It is cleaner and provides greater sexual pleasure.	Mixed	Malawi

VMMC, voluntary medical male circumcision; HIV, human immunodeficiency virus; STIs, sexually transmitted infections.

### Ethical considerations

Clearance was sought from the University of South Africa Health Studies Research Ethics Committee (reference numbers: HSHDC/881/2018 and MRCZ [MRCZ/A/2469]) before embarking on data collection through writing request letters as the research was initially intended to also cover human participants; the study, however, later focused on the narrative review. Even though there was no direct interaction with human subjects, there was still some possibility of some ethical concerns regarding human rights. According to Burns and Grove (2013:119), in concept analysis studies where the literature is reviewed, violation of human rights may be in the form of invasion of subjects’ privacy when reviewing documents. The researchers avoided misconduct in this research to ensure scientific integrity. Polit and Beck (2014:134) defined research misconduct as fabrication, falsification or plagiarism in proposing, conducting or reviewing research or in reporting results but does not include honest errors. Fabrication is defined as making up or forging of data or study results and reporting them and falsification is manipulation of materials or processes, or distorting results to give an inaccurate report of findings. Failure to acknowledge someone’s information or ideas is termed plagiarism (Polit & Beck 2014:134). Not only does misconduct cover the three types mentioned here but it also includes authorship improprieties, issues of conflict of interest and using confidential information without authorisation, among many other issues. In this study, in order to deal with issues of plagiarism, the researchers ensured accurate reporting of literature, avoided dishonesty and distortions and appropriately acknowledged through in-text citations and references.

## Findings

Four key categories emerged from the literature as follows:

1. Role of women

This was explained in terms of women being influential to men when they are knowledgeable enough. Women were also described as facilitators of couple communication and encouraging to men. They hold the negotiating and persuasive power and are able to insist men to go for MC.

2. VMMC uptake

Women are considered as strong support systems who prefer circumcised men, hence are able to promote VMMC uptake by men.

3. Barriers to VMMC

Barriers were found to be those factors that deter men from going for MC. The most common barriers identified were myths and misconceptions of MC, for example, the belief that men who go for VMMC become promiscuous. The other barriers were fear of pain, of an HIV test and fear of adverse events. These barriers need to be well addressed to reduce their effect so that men do not fear to go for MC and to educate women to convince men to take up the programme.

4. Facilitators of VMMC

Facilitators were identified as those factors that enhance VMMC uptake and the most common one was the belief that circumcised men take longer to ejaculate, and hence sexually satisfy their partners. Other facilitators observed were reduction of HIV, STIs, cancer of the penis and cervix and hygiene and attractive appearance of the penis. If well tackled, the facilitators can facilitate positive uptake of the VMMC programme.

## Discussion

The findings from the PICO model drew four main categories as follows: the role of women, VMMC uptake, barriers and facilitators of VMMC. It was found that women are key social systems whose important role is to influence men to undergo VMMC. According to Layer et al. (2014:258–272) and Reiss et al. (2014:e97748), encouragement of men by women was by either withholding sex or making VMMC a condition for establishing a sexual relationship. Women should use negotiating strategies that are in line with their relative position within relationships:

[*W*]omen can also be a source of information about MC for their male partners, and there is evidence that a woman’s preference for a circumcised partner can influence male interest in circumcision. (Baeten et al. 2010; Reiss et al. 2014)

This role is put as informational support by Cutrona and Suhr (1992:154–174) where women discuss with their husbands about MC and provide them more information about the importance of the procedure.

‘Women have been shown to influence and make decisions about whether their sons are circumcised and sway their male sexual partner’s decision to become circumcised’ (Rain-Taljaard et al. 2003:316). This is a very important role that can facilitate VMMC promotion, as long as women are knowledgeable enough.

Kobayashi (2014) supported by Reiss et al. (2014:e97748) stated that women may influence their male sexual partners’ decision to be circumcised. The women felt they influenced men to be circumcised by talking to them about circumcision or by insisting that they are circumcised. The researches outlined the roles of women after empowerment by the healthcare workers as creating awareness of VMMC, counselling of men on MC, influencing and swaying their sexual partners and male children to utilise MC. The women also encourage men to go for MC, educate men and generally act as the source of VMMC information for men. Five social support categories were outlined to guide the women in influencing men’s perceptions.

Factors were observed to be either enhancers or barriers to VMMC. The PICO approach drew main elements of social support systems, focusing specifically on women as the key social support systems, promoting VMMC uptake, which is the intervention. According to Nxumalo and Mchunu (2019:9–17), the degree of knowledge, coupled with the females’ acceptance of the procedure, facilitates women to be able to act as motivators for circumcision. This means that the more knowledgeable women are, the more acceptable they become of the process, hence promoting the uptake of VMMC by men. An interesting finding from various researchers was the benefit of VMMC in improving sexual pleasure and attractiveness of the penis. This is believed to encourage many men and women to consider VMMC in order to enhance improved relationships. The benefits of MC act as enhancers to VMMC uptake, hence can be taken as the outcome in the PICO model.

The PICO component of comparison was related to barriers to MC causing reduced VMMC uptake. Taruvinga (2014) stated the barriers as fear of pain, myths and misconceptions about VMMC (infertility), men think that they will not catch HIV so they feel they do not need to go for MC and partner refusal and fear of an HIV test. If these barriers are not adequately addressed, they will deter many women and men, which results in a negative attitude towards VMMC.

The concepts identified in the literature and linking the elements were change agent, recipients, role of women and outcome. The change agents were identified as the healthcare workers responsible for empowering the recipients, women. The involvement of women in VMMC was also discussed at length, and it was found that it is a vital recommendation from most researchers and is believed to improve uptake of VMMC. Majority of the studies concur on the importance of involving social support systems in VMMC uptake. A rich base of knowledge on VMMC and social support was identified, thus highlighting limited studies performed on women as social support systems. Identified literature related to variables guided formulation of research tool.

Healthcare workers are to realise that promoting MC is a complex approach, which needs vigilance and multidisciplinary approach in terms of utilisation of various social support systems, not only women.

Healthcare workers in VMMC must be well supported and trained in effective demand creation to promote uptake of VMMC, this could be ensured by conducting workshops for women, especially at churches and women’s clubs, to empower them with knowledge and skills on MC and negotiating strategies to their partners.

If people are equipped with enough knowledge on MC, they are able to make informed decisions on whether they should take up the services. The researchers recommend that couple communication need to be taught between couples to enable them to discuss health-related issues.

## Limitations

The use of broad integrated literature search for concept formulation could have led to some important aspects on MC and social support systems to be missed. It is, however, hoped that because MC is a topic that has been widely researched, most major key concepts on MC and social support systems, specifically women, were captured.
